# Sites Inferred by Metabolic Background Assertion Labeling (SIMBAL): adapting the Partial Phylogenetic Profiling algorithm to scan sequences for signatures that predict protein function

**DOI:** 10.1186/1471-2105-11-52

**Published:** 2010-01-26

**Authors:** Jeremy D Selengut, Douglas B Rusch, Daniel H Haft

**Affiliations:** 1 J. Craig Venter Institute, 9704 Medical Center Drive, Rockville, MD, USA, 20850

## Abstract

**Background:**

Comparative genomics methods such as phylogenetic profiling can mine powerful inferences from inherently noisy biological data sets. We introduce Sites Inferred by Metabolic Background Assertion Labeling (SIMBAL), a method that applies the Partial Phylogenetic Profiling (PPP) approach locally within a protein sequence to discover short sequence signatures associated with functional sites. The approach is based on the basic scoring mechanism employed by PPP, namely the use of binomial distribution statistics to optimize sequence similarity cutoffs during searches of partitioned training sets.

**Results:**

Here we illustrate and validate the ability of the SIMBAL method to find functionally relevant short sequence signatures by application to two well-characterized protein families. In the first example, we partitioned a family of ABC permeases using a metabolic background property (urea utilization). Thus, the TRUE set for this family comprised members whose genome of origin encoded a urea utilization system. By moving a sliding window across the sequence of a permease, and searching each subsequence in turn against the full set of partitioned proteins, the method found which local sequence signatures best correlated with the urea utilization trait. Mapping of SIMBAL "hot spots" onto crystal structures of homologous permeases reveals that the significant sites are gating determinants on the cytosolic face rather than, say, docking sites for the substrate-binding protein on the extracellular face. In the second example, we partitioned a protein methyltransferase family using gene proximity as a criterion. In this case, the TRUE set comprised those methyltransferases encoded near the gene for the substrate RF-1. SIMBAL identifies sequence regions that map onto the substrate-binding interface while ignoring regions involved in the methyltransferase reaction mechanism in general. Neither method for training set construction requires any prior experimental characterization.

**Conclusions:**

SIMBAL shows that, in functionally divergent protein families, selected short sequences often significantly outperform their full-length parent sequence for making functional predictions by sequence similarity, suggesting avenues for improved functional classifiers. When combined with structural data, SIMBAL affords the ability to localize and model functional sites.

## Background

Phylogenetic profiling is a powerful discovery method in bioinformatics. In this method, typically, the presence or absence of a member of a protein family in a genome is treated as a trait whose phylogenetic distribution can be compared to that of another trait, usually meaning another protein family. The joint presence or joint absence of two traits over a sufficiently large and varied set of species provides strong statistically-based evidence that those traits are functionally connected in some fashion [1]. Profile methods are being used increasingly to relate protein families to varied types of second traits such as phenotype, biological niche, transcriptional regulatory sites, and so on [2]. One such type of trait, metabolic capability, can be calculated by the Genome Properties system [3] using rules based largely on hidden Markov Models (HMMs) from the TIGRFAMs collection [4], as well as by the application of other methodologies such as Subsystems [5] or MetaCyc [6]. For example, one can determine which species have and which lack the capability to synthesize menaquinone or metabolize urea, even when those capabilities are encoded in different ways by different organisms. We have found that these assertions of metabolic background (profiles) provide excellent opportunities for launching phylogenetic profiling studies.

Phylogenetic profiling (PP) methods often are limited by their reliance on pre-constructed protein families, or on fixed parameters that serve in lieu of pre-constructed families. Recently, we addressed this limitation by introducing Partial Phylogenetic Profiling (PPP) [7]. PPP uses a given phylogenetic profile as a query, and determines which proteins in a target genome score best against that profile. Each protein is scored by selecting the (BLAST) sequence similarity cutoff that optimizes its match to the query profile. As the algorithm explores more and more permissive cutoffs, more genomes are added to the set of genomes compared to the query profile. At each depth, this set corresponds to only a part of the query profile (hence "partial"), and the preponderance of matches over mismatches is scored according to the binomial distribution. For each protein, the depth at which the agreement with the query profile scores best, and the score itself, are recorded. Once all genes in a genome have been run, sorting by score reveals those proteins that best match the profile. The on-the-fly optimization method eliminates dependence on pre-defined protein families. It enables profile-based discovery even where pre-built families are lacking, and obviates the need for pre-set score thresholds.

Profiling methods may indicate strongly that some biological connection underlies the regular co-occurrence of two traits, but they do not always reveal the nature of the connection. PPP and other profiling methods provide inferences about the functional relationships between full-length proteins and the biological systems represented by query profiles. A biochemical basis for those relationships might be revealed by identifying which specific protein sequence domains and motifs are most responsible for the correlation of two traits. Therefore, a computational method to dissect and explain the origin of PPP's signal may provide a wealth of additional clarity and insight, especially when different sites within a protein mediate interactions with substrates, cofactors, and/or auxiliary proteins.

The basic scoring mechanism from PPP - using binomial distribution statistics to optimize sequence similarity cutoffs during searches of partitioned training sets - can be reused to discover key subsequences in groups of proteins. Here we demonstrate that SIMBAL, Sites Inferred by Metabolic Background Assertion Labeling, generalizes that approach by providing the additional freedom to apply phylogenetic profiling methods locally within a protein sequence. We show that SIMBAL can mine a protein sequence for short sequence regions, presumably containing critical sites, and that it outperforms other simple classifiers, such as BLAST matches to full-length proteins, for the task of classifying functionally diverged members of homology families.

## Results

### Training sets classified by metabolic context: urea ABC transporters

**Figure 1:**
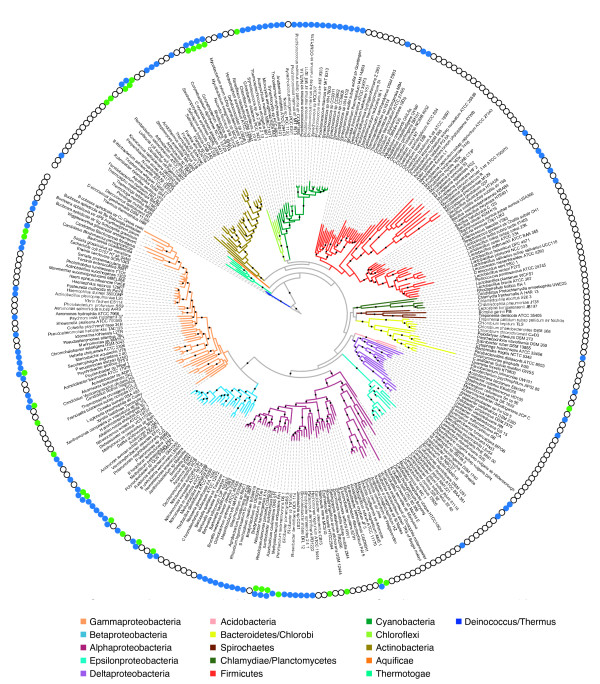
**The phylogenetic distribution of urea metabolism in bacteria is broad and sporadic**. The urease system (GenProp0051, blue circles) and the urea carboxylase/allophanate hydrolase pathway (GenProp0481, green circles) are plotted on the AMPHORA inferred phylogenetic tree of prokaryotes from Eisen & Wu[24]. Certain archaeal species also metabolize urea and are not shown (although they were included in the analyses).

Several features of urea transporter permeases make them attractive for demonstrating the potential of SIMBAL. Functional prediction for transporter proteins, important as it is, is difficult because these proteins are highly hydrophobic and may be difficult to study by crystallographic techniques. Similarly, these transporters show relatively weak sequence conservation, complicating inferences made solely from pairwise homology comparisons. However, urea utilization and urea uptake both are broadly and sporadically distributed (Figure 1), giving strong, clear signals for comparative genomics methods. The multi-component systems that are required for urea-utilization, especially the nickel cofactor-dependent urease system that numerically dominates over the urea carboxylase system, provide clear discrimination between utilizers and non-utilizers. Independent characterizations of urea transport operons in Cyanobacteria (*Synechocystis sp*. PCC 6803 and *Anabaena sp*. PCC 7120) [8] and Actinobacteria (*Corynebacterium glutamicum*) [9] reveal considerable sequence divergence across species for the corresponding permease subunits, including substantially different lengths, despite their shared function. In a large number of distinct lineages (*Burkholderia malei, Ralstonia eutropha, Bradyrhizobium japonicum, Deinococcus radiodurans, Prochlorococcus marinus, Bacillus halodurans, Haloarcula marismortui, etc*.), the urease operon and urea ABC transporter operon are adjacent, providing additional support through local gene context for extending the set of trusted urea transporter sequences. Having five subunits in the urea ABC transporter (the substrate-binding protein UrtA, permease subunits UrtB and UrtC, and ATP-binding cassette proteins UrtD and UrtE) provides a means to test the self-consistency of the transitive annotation of function from one genome to another. All belong to larger families that have considerable numbers of homologs of differing function (as inferred from their large numbers of paralogs with differing current public annotations) from which they must be discriminated. The remote but real homology between the two permease subunits UrtB and UrtC (below 25% even though both proteins share the reduced complexity from being highly hydrophobic integral membrane proteins) provides an opportunity to examine similarities and differences in the locations of the apparent hot-spots of predicted functional specificity. Therefore, the pair of permease subunits of the urea ABC transporter was chosen as the first test system for SIMBAL.

### Partial Phylogenetic Profiling (PPP)

The Genome Properties rule for urea utilization (see Methods) was used to generate a profile for Partial Phylogenetic Profile (PPP) analysis. Urea utilization was taken as a union of species with urease and those with the urea carboxylase pathway. PPP of the genome of *Corynebacterium glutamicum *vs. this profile revealed, as the top eleven hits, six (out of seven) components of the urease system, followed by the known five-gene operon for a urea ABC transporter. All scores exceeded that of the (non-urea related) twelfth hit by more than three orders of magnitude. The results, shown in Table 1, display the power of phylogenetic profiling methods for associating proteins with biological processes and molecular functions. PPP applied to other genomes with putative urea ABC transporters similarly found complete five-gene operons in the top tier of results, confirming predictions made by TIGRFAMs "equivalog"-level HMMs: TIGR03407 (substrate binding protein UrtA), TIGR03409 and TIGR03408 (permease subunits UrtB and UrtC), and TIGR03411 and TIGR03410 (ATP-binding cassette subunits UrtD and UrtE).

**Table 1:** Top scoring Partial Phylogenetic Profiling results for urea utilization vs. *Corynebacterium glutamicum *ATCC 13032, based on 117 TRUE genomes and 235 FALSE genomes.

PPP score^a^	TRUE	Depth	Locus	Protein
45.4	95	95	NCgl0083	urease, gamma subunit UreA

45.4	95	95	NCgl0085	urease, alpha subunit UreC

45.4	95	95	NCgl0088	urease accessory protein UreG

44.9	94	95	NCgl0084	urease, beta subunit UreB

39.2	82	82	NCgl0087	urease accessory protein UreF

34.9	73	73	NCgl0089	urease accessory protein UreH

28.5	66	68	NCgl0894	urea ABC transporter, permease subunit UrtB

28.2	59	59	NCgl0895	urea ABC transporter, permease subunit UrtC

26.9	65	68	NCgl0893	urea ABC transporter, substrate-binding UrtA

24.1	59	62	NCgl0896	urea ABC transporter, ATPase subunit UrtD

19.5	55	61	NCgl0897	urea ABC transporter, ATPase subunit UrtE

a) PPP scores are reported as the optimal negative log odds against observing the indicated number of hits to the TRUE profile at the indicated search depth.

### SIMBAL: using metabolic background assertions to find key subsequences

**Figure 2:**
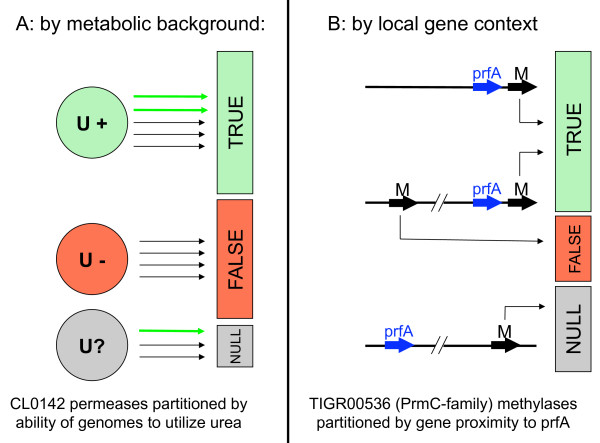
**Methods for partitioning a protein family for SIMBAL analysis**. A) Partitioning by metabolic background assertions: members are assigned to the TRUE branch based on the observed presence of a metabolic pathway (or markers thereof) in their parent genomes (here, the ability to metabolize urea). The TRUE branch will contain both members involved in the chosen metabolism (green arrows) and, if there are many family members per genome, members which are not (black arrows). The FALSE partition will have few mis-attributed members. B) Partitioning by local genomic context: members are assigned to the TRUE branch by virtue of their proximity to a chosen marker (here, the substrate of a particular methylase among several in the larger family), while other members from the same genome are assigned to the FALSE branch. In genomes where no proximity cues are present members are omitted from the analysis. While not applicable to the PrmC example, one could also assign to the FALSE partition based on the proximity of members to markers of functions other than the one of interest.

A training set of sequences from PF02653 ("Branched-chain amino acid transport system/permease component") and from all other families in the same Pfam [10] clan, CL0142 ("Membrane_trans"), were collected from the CMR [11], partitioned based on apparent urea metabolism potential of the source genomes, made non-redundant at 80% sequence identity, and used for SIMBAL analysis (see Figure 2A and Methods). This training set consists of 5224 sequences in the TRUE branch and 4887 sequences in the FALSE branch.

**Figure 3:**
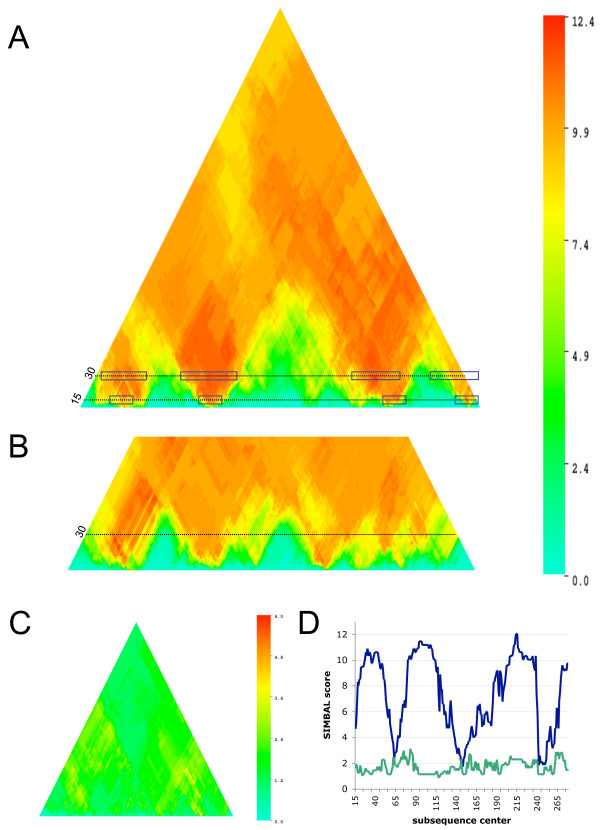
**SIMBAL results on ABC permeases versus a urea metabolism partition of the Pfam CL0142 clan**. A) SIMBAL triangle heat map for the *Corynebacterium glutamicum *UrtB protein (NCgl0894) constructed of stacked rows of output from windows of size 5 amino acids up to the full length of the protein, colored by the SIMBAL score. Peaks at window sizes 15 and 30 are indicated in blue boxes. B) A portion of the heat map for the *C. glutamicum *UrtC protein (NCgl0895). C) A heat map for the non-urea transporting *C. glutamicum *NCgl0030 permease at exaggerated scale illustrating negligible response. D) SIMBAL score plot of UrtB (blue) and NCgl0030 (green) at a window size of 30 amino acids.

The genome of *Corynebacterium glutamicum *ATCC 13032 encodes all structural and accessory components of the urease system [9], so all 23 *C. glutamicum *members of the CL0142 clan are assigned to the TRUE branch of the SIMBAL training set (Figure 2A). This includes the urea ABC transport permease subunits NCgl0894 (UrtB, [GenBank:BAB98324]) and NCgl0895 (UrtC, [GenBank:BAB98325]), members of PF02653, which have been characterized [9], are clustered with the urease genes and are identified as top hits by PPP analysis. Results of SIMBAL analysis are plotted in Figure 3. Each subsequence is designated by the location of its center on the parent sequence (x-axis) and by its length (y-axis), and then given a color to show its SIMBAL score. Results are presented as heat maps, where hot spots in red indicate the most significant SIMBAL scores while blue shows the weakest. Because larger sequence windows have less freedom to slide, the resulting graphic is triangular in shape. The apex represents the greatest possible subsequence length, equal to the full length of the protein, with its center at the midpoint along the protein sequence. These plots clearly indicate that, in this protein family in *C. glutamicum*, the two *bona fide *urea ABC transporter permease subunits have significant regions of relatively high score. The results also show large regions where the sequence seems to contain hardly any evidence of the parent protein's specificity for urea transport. Notably, the best performing subsequences ("hot spots") for these two permeases outscore their respective full-length parent sequences. In contrast, the three other *C. glutamicum *members of this family are nearly featureless indicating that no portion of these sequences, when used as a BLAST query succeeds in recovering the pattern of urea utilizing species. One of these, NCgl0030 (GenBank:BAB97424], is shown as a triangle plot at exaggerated scale in Figure 3C and as a direct comparison of the size-30 subsequences in Figure 3D.

**Figure 4:**
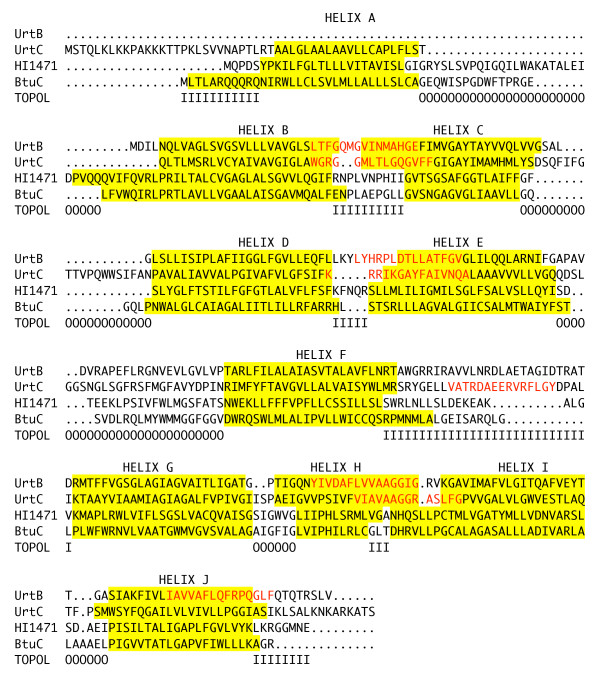
**Structural alignment of crystallized ABC permeases with UrtB and UrtC**. Primary sequence of HI1471 from *H. influenzae *and BtuC from *E. coli *were aligned by MUSCLE. The ten transmembrane helices (A-J) observed in the respective structures are indicated in yellow. Predictions of transmembrane helices of *C. glutamicum *UrtB and UrtC were carried out by MEMSAT3[14] and are also shown in yellow. The UrtB and UrtC sequences were added to the HI1471/BtuC alignment manually. Topology of the crystallized permeases is indicated in the last line (O:outside, I:inside). The four highest scoring SIMBAL subsequences at 15 amino acid length for UrtB and UrtC are indicated in red font. Note that all are partially or completely localized to the predicted inside (cytoplasmic) face of the membrane.

**Figure 5:**
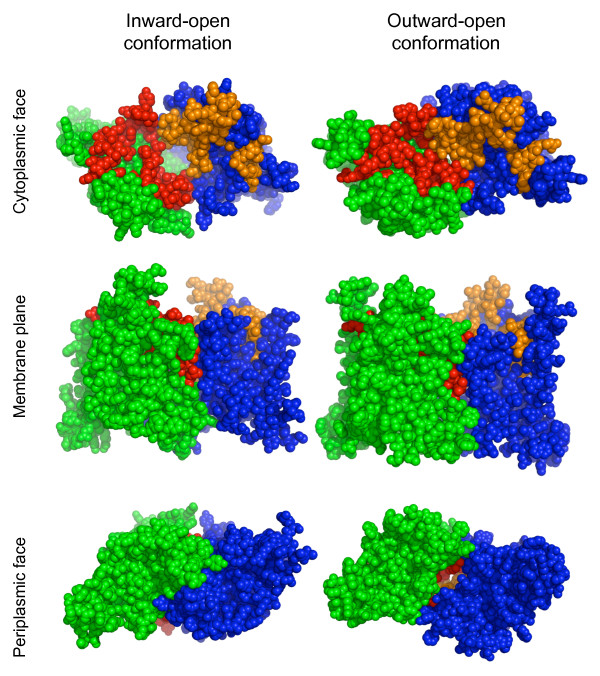
**Structural mapping of UrtBC SIMBAL hotspots**. Left: Mapping onto the "inward-open" structure of HI1471 [PDB:2NQ2][13]. Right: Mapping onto the "outward-open" structure of BtuC [PDB:1L7V][12]. SIMBAL hotspots are clustered around the inward-facing pore. In each example the homodimeric structures are shown in blue and green, with one subunit (blue) representing UrtB, and the other (green) representing UrtC. Hotspots are shown in red and orange, respectively. Molecular models visualized with MacPyMOL http://www.pymol.org/.

Although no crystal structures have been determined for UrtBC, two structures from homologous transporters in the CL0142 clan have been published: the B12 transporter permease/ATPase complex BtuCD from *E. coli *[PDB:1L7V] [12], and the unknown specificity permease/ATPase complex HI1471/HI1470 from *Haemophilus influenzae *[PDB:2NQ2] [13]. Both of these structures include single-gene, homodimeric permeases, in contrast to the heterodimeric UrtBC. Notably, these two structures capture two different conformational states of the permease, inward-open in the case of HI1471 and outward-open in the case of BtuC. These two permeases are more closely related to one another than either is to UrtBC. It is difficult to calculate a reliable sequence alignment between UrtBC and either of these reference sequences due to their overall divergence (UrtB and HI1471 have less than 20% aligned identity, for instance). Secondary structure prediction algorithms can be utilized to derive the approximate location of the transmembrane helices of UrtB and UrtC and these in turn used to constrain a multiple sequence alignment. We utilized the program MEMSAT3 [14] and found it to yield consistent results when applied to UrtBC, BtuC and HI1471 with little disagreement as to the position and number of membrane-spanning helices. The only major structural difference between UrtBC and the crystallized permeases is the lack of the N-terminal helix in UrtB (Figure 4). Based on this alignment, the SIMBAL hotspots from UrtBC can be mapped onto the corresponding segments of the crystal structures. Even allowing for some error in the alignment, it is clear that SIMBAL only identifies residues on the cytoplasmic face of the permease, and that the first two peaks in both UrtB and UrtC identify every residue of the exit pore in the inward-open conformation (Figure 5).

This observation suggests that, for UrtB and UrtC-like ABC transporter permease subunits, the most prominent evolutionary constraints tied to urea utilization are neither interactions with the substrate-binding protein nor with the substrate where it begins to transit the membrane. Rather, the key sites that track with urea utilization appear to be those that control release of the substrate on the cytosolic face of the plasma membrane. Exit from the channel corresponds to a change in transporter complex quaternary structure as part of the cycle that couples ATP hydrolysis to substrate translocation [15].

The evolutionary history of UrtB and UrtC-like transporter permeases clearly contains numerous examples of paralogy formation and neofunctionalization, such that the protein family now contains examples of both ancient divergences with conserved function, and more modern splits with divergent function. By marking several small regions, separated along the main chain but close in the folded structure and surrounding the pore, SIMBAL shows that transporter permease subunits indeed contain sequences that tend to predict functional specificity rather than recent common ancestry.

The genome of *Bradyrhizobium japonicum *USDA 110 encodes 78 CL0142 ABC transporter permease domains, 73 of which are in the same PF02653 family as UrtBC. These are organized in 45 distinct clusters, 27 of which are of the 5-gene type (binding-protein, permease, permease, ATPase, ATPase) typical of the known urea transporter. A phylogenetic tree of the PF02653 permeases (not shown) has 4 major branches that can be classified as UrtB-like, UrtC-like, sugar transporter-like (3 and 4-gene types) and unknown (4-gene type). All 78 of these were analyzed by SIMBAL. Only certain members of the UrtBC-like clades showed any peaks by SIMBAL above noise. Two of these genes (blr1449 [GenBank:BAC46714] and blr1450 [GenBank:BAC46715]) reproduce the pattern of hotspot peaks observed for the *C. glutamicum *UrtB and UrtC genes and are in fact the *B. japonicum *homologs of UrtB and UrtC that are observed adjacent to the genes for the urease enzyme system. An additional pair of genes (blr0968 [GenBank:BAC46233] and blr0969 [GenBank:BAC46234]) have SIMBAL peaks nearly indistinguishable from those of the urease-linked permeases in *C. glutamicum *and *B. japonicum*. Interestingly, this second 5-gene ABC transporter cluster is adjacent to a gene of the PF03069 Acetamidase/Formamidase family (blr0972, [GenBank:BAC462337]) and a gene of the PF00795 carbon-nitrogen lyase family annotated as "aliphatic amidase, AimE" (blr0973, [GenBank:BAC462338]), strongly suggesting that the transported molecule is not urea, but some other amide-containing molecule or molecules, possibly acetamide or formamide.

The examination of SIMBAL results for *B. japonicum *provides a cautionary clarification. The hot-spots identified as highly similar in both raw sequence and SIMBAL score still should be interpreted to reflect functional specificity that reflects substrate chemical properties. This specificity, however, may include related amides, echoing the ability of urea carboxylase from *Oleomonas sagaranensis*, for instance, to act on formamide and acetamide as well as urea [16].

### SIMBAL applied to training sets classified by local genomic context

Local genomic context can provide strong clues to protein function. The S-adenosylmethionine-dependent protein-(glutamine-N5) methyltransferase PrmC (HemK) [17] N-methylates a glutamine residue in a specific Gly-Gly-Gln motif of peptide chain release factors 1 and 2 (RF-1, RF-2; products of the *prfA *and *prfB *genes)[18]. *PrmC *and *prfA *are adjacent in Escherichia coli and many other bacterial species. A complex of PrmC with RF-1 has been solved crystallographically [17], and can provide a context for interpreting results from SIMBAL analysis.

Candidate PrmC proteins were identified by using the broad-specificity TIGRFAMs model TIGR00536. This model represents a family of methylases including genuine PrmC proteins, a number of unidentified methylases and the E. coli YfcB protein, identified as the methylase (PrmB) carrying out the glutamine methylation of ribosomal protein L3 [19]. Hits to the TIGR00536 model were placed in the TRUE partition for the PrmC SIMBAL training set (Figure 2B) if they were encoded in the immediate neighborhood of *prfA *(at most two intervening genes). In species with two paralogous PrmC family proteins, the one not encoded near *prfA *was placed in the true-negative set. In species with a *prmC *homolog that was not near to *prfA*, the protein was not used in either partition of the training set since it is not possible to conclude whether the gene is active on one, both or neither of RF-1 and L3. Both RF-1 and L3 themselves are universal in all bacteria, but their post-translational modifications may not be. Each partition was made non-redundant to no more than 80% sequence identity, and SIMBAL analysis was performed. This training set contains 187 proteins in the positive branch and 62 proteins in the negative branch. Note that this training set is 1/40^th ^the size of that used in the previous example and has a 3:1 true-false ratio as opposed to the 1:1 ratio used previously. This training set, though smaller, should contain essentially no false positives (noise) by virtue of the way it was constructed as opposed to the considerable amount included in the permease example. Also of notable difference is the much less sporadic nature of the PrmC profile.

In theory, SIMBAL should detect short sequences that outperform full-length sequences for making functional predictions by BLAST versus members of the homology family under study. This will be observed primarily when neofunctionalization has occurred within the clade containing the target function. In such a case, better functional predictions are made by conservation at critical sites than by time since the most recent common ancestry. Because neofunctionalization events happen sporadically, some sequences will show SIMBAL hot spots in much starker contrast to the apex (full-length sequence) signal than do others. Comparing results based on several different starting sequences, however, adds a measure of confidence through consistent "voting" for functionally important sites.

**Figure 6:**
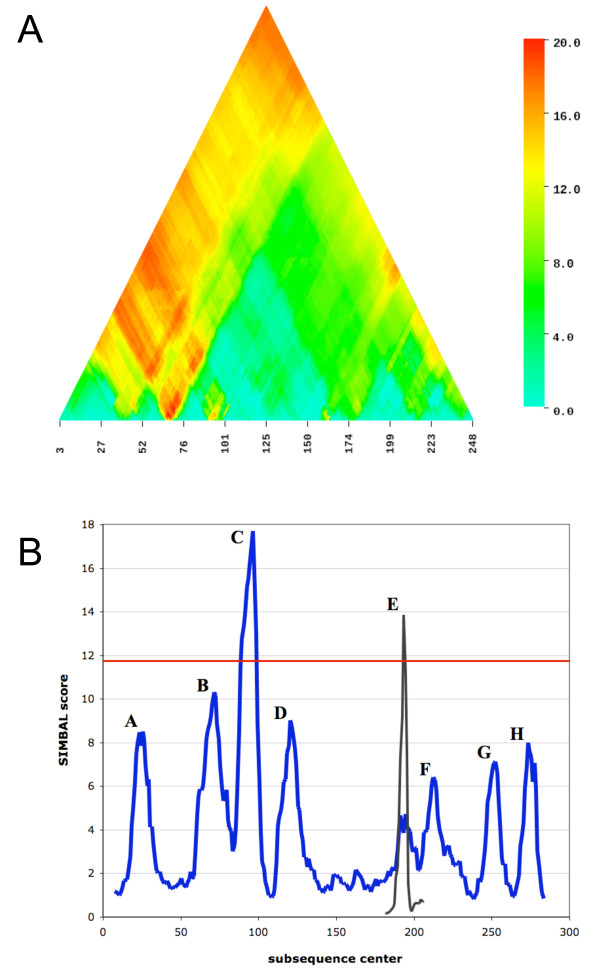
**SIMBAL results on PrmC RF-1-specific methylases versus a PrmC/RF-1 proximity partition of the TIGR00536 PrmC-like methylase family**. A) SIMBAL triangle heat map for the *Bordetella bronchiseptica *RB50 PrmC protein (BB0385, [GenBank:CAE30883]). B) A SIMBAL score plot of 10 PrmC proteins averaged after local registration of the SIMBAL hotspots by alignment of the corresponding subsequences, at a window size of 14 (blue) and, in the vicinity of peak E, at 6 amino acids (black). The average SIMBAL score for the full-length proteins is shown in red. PrmC sequences came from the genomes of: *E. coli *536, *Arthrobacter aurescans*, *Bacillus clausii *KSM-K16, *Mycoplasma hyopneumoniae *232, *Mesoplasma florum*, *Sinorhizobium meliloti *1021, *Zymomonas mobilis *ZM4, *Rhodococcus *sp. RHA1, *Desulfitobacterium hafniense *Y51 and *Bordatella bronchiseptica *RB50.

SIMBAL analysis was first performed against the sequence of the (crystal structure solved) PrmC protein from *E. coli *W3110 (b1212, [PDB:1T43]). This protein is a member of the TIGR00536 PrmC family and has been characterized as the methyase acting on RF-1 and RF-2 in vitro [19]. This SIMBAL plot is dominated by five major peaks with scores in excess of 15, and several additional minor peaks with scores between 5 and 15. Sequences of members of the PrmC TRUE partition were aligned and confirmed to form a single clade by phylogenetic analysis (not shown). Ten sequences were chosen to represent diverse subclades of this tree and each was analyzed by SIMBAL. We observed some variation in the number, relative position and strengths of peaks. A typical result is illustrated in Figure 6A. To obtain a consensus, all of the SIMBAL results at window sizes of 14 and 6 amino acids were co-registered by locally aligning the subsequences for each peak and were then averaged (Figure 6B). The average value for the full-length sequences (plot apex) is also indicated.

**Figure 7:**
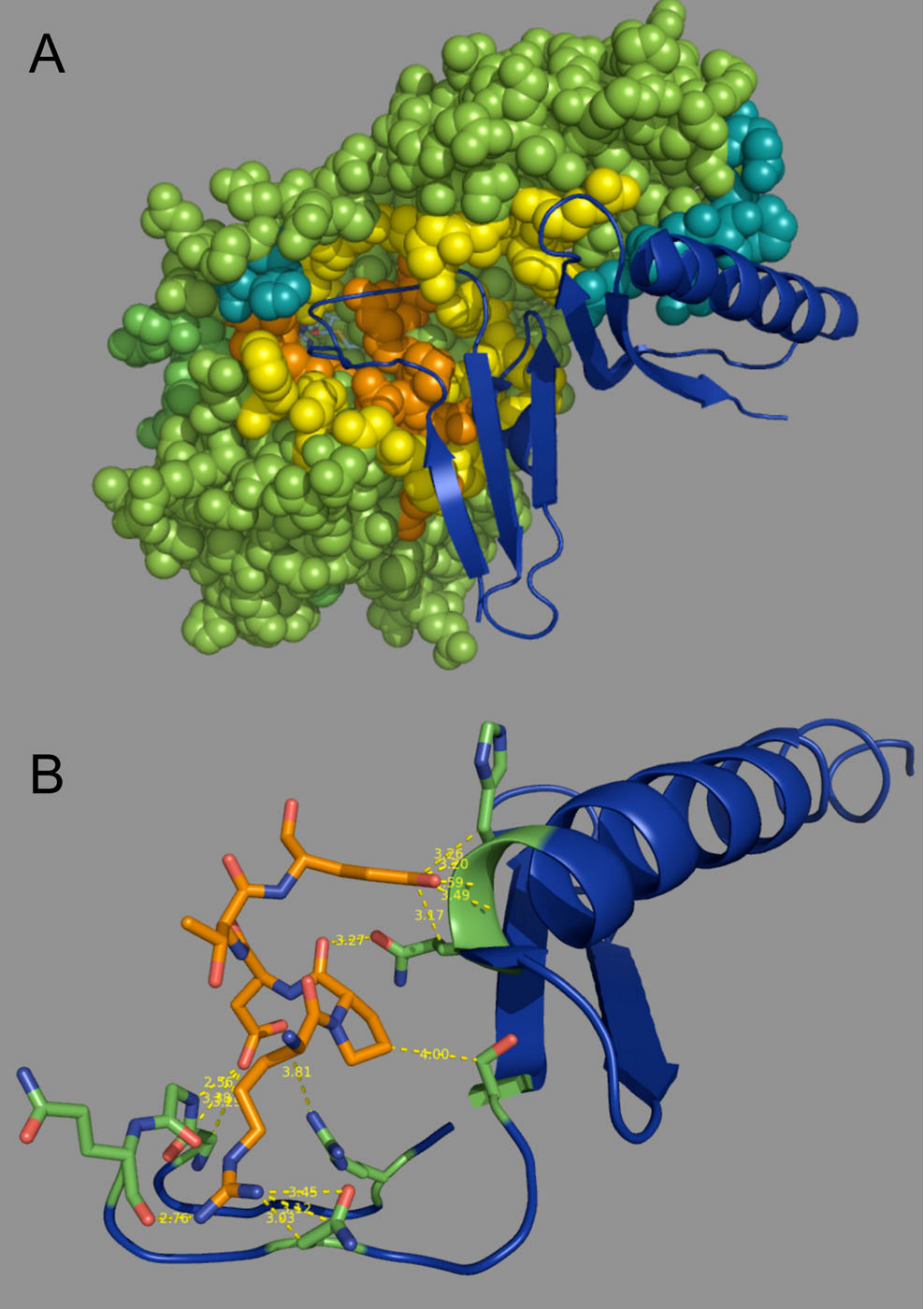
**Structural mapping of PrmC SIMBAL hotspots**. A) PrmC is shown as a space-filling model. Subsequences C and E are shown in orange, others in yellow. Deeper colors indicate residues in contact with the RF-1 substrate. Contact residues not identified by SIMBAL are shown in blue-green. All other PrmC residues are in green. Segments of RF-1 domains 2 and 3 in contact with PrmC are shown as a ribbon cartoon in blue. The SAM cofactor is shown at the bottom of the active site cleft as a stick model. B) A detail of the contacts made by the RPDTE motif of subsequence C. PrmC is shown in orange, contacted residues of RF-1 are in green, otherwise colored blue. Molecular models visualized with MacPyMOL http://www.pymol.org/.

The eight subsequences identified by SIMBAL (Table 2) include 33 of the 42 residues observed to make contacts with RF-1 in the complex crystal structure [19]. Of the nine not identified by SIMBAL, seven are the most distal from the active site and make contact with an RF-1 domain distinct from that containing the methylation target glutamine (Figure 7A). Subsequences C and E, displaying the strongest SIMBAL scores, are positioned closest to the active site and make contacts with the residues flanking the conserved GGQ target sequence and the target motif itself. Subsequence C, in particular, dominates the SIMBAL results and is observed as the strongest peak in every sequence analyzed. One may infer that this subsequence encodes the most distinctively PrfC-like portion of the protein, mediating not the catalysis *per se*, since other PrfC-homologs presumably use the same catalytic mechanism, but the discrimination of the substrate from other potential methylation targets. Unlike all the other susbsequences identified, subsequence C appears to have a central position in the binding surface, its RPDTE motif making contacts with RF-1 at the methylation site and the sequence loops immediately before and after, as well as positioning a negatively charged group (Glu-96) at the positive dipole end of the following alpha helix (Figure 7B).

**Table 2:** SIMBAL-derived subsequences from PrmC.

Subsequence^a^	*E. coli *PrmC subsequence^b^	Consensus^c^	SIMBAL score^d^
A	18-**ES**P**RRD**AEI**L**LEHV-31	esprlDAel**L**Lahv	8.5

B	66-EP**I**AHLTGV**REFW**S-79	E**P**vaY**I**l**G**erE**F**wG	10.3

C	90-**I**P**RPD**T**E**CLV**E**QAL-103	I**PR**PD**TE**eLVEaaL	17.7

D	115-DLGTGTGAIALALA-128	**D**LG**TG**S**G**AIAlALA	9.0

E	182-S**NPPYI**-187	S**NPPY**I	13.8

F	197-G**DV**RFEPLT**AL**VAA-210	eVlrfE**P**rs**AL**faG	6.4

G	237-EH**GW**QQGEAVRQAF-250	**E**i**G**yd**Q**geaVraLf	7.1

H	259-ETCRD**YG**D**N**E**R**VTL-272	etrk**D**LaGnd**R**vvl	7.9

a) subsequences as indicated in Figure 6B. b) Contacts with RF-1 observed in the PrmC:RF-1 crystal structure indicated in bold. c) Lower-case residues are less than 50% conserved, residues in bold are greater than 80% conserved. d) Scores are reported at a window of 14 amino acids except for subsequence E which has a window size of 6.

SIMBAL does not identify merely the most conserved motifs in a protein family; subsequences D and E are equally well conserved as the high-scoring subsequence C (Table 2). Rather, what are found are those regions conserved enough that BLAST identifies them efficiently within the TRUE partition of the family, yet divergent enough that corresponding sequences outside the desired partition are excluded. In the case of subsequence C, the difference between the consensus from the positive and negative branches of the partition is clear:

The sequences are variable precisely at the place where the most significant substrate contacts are made (Figure 7B). In comparison, subsequence D, which contains the well-conserved SAM-methylase motif I [20] is not only conserved among the true PrmC genes, but is only slightly different in the PrmC-like proteins. This is consistent with its position adjacent to the SAM cofactor where it makes no direct contacts with the substrate.

Displaying the full heat map (Figure 6A), rather than just a SIMBAL trace for a fixed window size, shows an interesting attribute for motif C. Not only is it the strongest of the peaks at the selected window size, but its signal remains conspicuous in longer and longer windows (points plotted higher up in the triangular heat map) as long as they include the motif. This behavior creates a feature in the SIMBAL heat map in which a large red and yellow area appears to spread upward in a V-shaped "plume" from a highly localized single site, the center of motif C. Features of this type likely represent both the strength of the signal at small window sizes and supporting signal from neighboring residues. However, the mapping of SIMBAL peaks from short window sizes to crystal structure shows that even sites that lack such V-shaped plumes in the heat map remain important for finding and interpreting determinants of protein functional specificity.

## Discussion

In developing the PPP method [7] we introduced the strategy of using BLAST results from every gene in a genome and scoring the results based on the likelihood that the top hits would predominantly hit genes from the genomes in a given phylogenetic profile. Here, in the SIMBAL method, we have transferred that methodology to an analysis of individual protein sequences. Instead of scanning every gene in a given genome, we instead scan all subsequences of a given protein sequence. BLAST is again utilized, this time versus all proteins in the same homology family where that family has been partitioned by some method. This binary partition of the data is analogous to a phylogenetic profile and may, as in the urea permease example, be derived directly from such a profile. The SIMBAL method is generic, however, in the sense that any method may be applied for the separation of the proteins into TRUE and FALSE groups, discriminating among homologs from the same genome as in the PrmC example where this makes sense. The interpretation of SIMBAL results will depend directly on the biological relevance of the partitioning rationale.

In this work, in order to focus on the methodology itself, we have chosen examples where the results could be verified versus solved crystal structures with published analyses and interpretations, where much is known about the relevant biochemistry, where the proper annotation of gene function is relatively secure and the understanding of subunit architecture and key functional sites is fairly advanced. Nevertheless, we did not use any of this information *a priori*, bit relied instead on proxies for constructing the protein family partitions such as might be used in circumstances where far less is known about the proteins to be analyzed.

In the urea permease example, many urea ABC transporters are present in clear-cut operons near genes for the catabolism of urea making their annotation straightforward.

The partition method we chose, however, is only based on the metabolic potential of the genomes, and results in the incorporation of large numbers of non-urea permeases into the TRUE branch of the partition. Despite this "noise" in the dataset, SIMBAL was able to give unambiguous results for relevant permeases. This robust behavior is due in part to the high information content of the urea metabolism profile, but also can be traced to the algorithm itself which only scores top BLAST hits to the TRUE branch relative to the number of hits encountered so far in the hits list, and is agnostic to the size of the TRUE set and the proportion of that set which has been encountered. Clearly, had there been no information about which permeases were involved in urea transport (only the informed guess that ABC transporters were involved) SIMBAL could have been iterated and used like PPP, scanning every member of the relevant protein family for strong signals (as was done here with the *B. japonicum *permeases).

In the PrmC example, we chose to construct the partition based solely on local prmC-prfA operonic context and the inference that additional PrmC homologs in those genomes were not PrmC. This partition results in a training set far smaller than it could have been if other information had been used, and susceptible to bias based on the non-random distribution of operonically clustered family members. Despite these self-imposed drawbacks, SIMBAL derives a list of subsequences covering most of the PrmC-RF-1 contact surface and clearly identifying the crucial substrate-binding motif.

Some noise is inherent in these analyses, from the small sample size of sequence regions, anisotropy of molecular evolution, and quirks of scoring sequence similarity by BLAST. However, the graphical display of scores obtained by SIMBAL shows relatively smooth curves with clear peaks, rather than scores differing noisily over the length of the sequence. The signals appear to be real, well-behaved, and consistent with PPP analysis and crystal structures.

We have observed a decided advantage of certain subsequences in these examples to outperform the full-length protein sequences in BLAST-based discrimination of the TRUE and FALSE branches of the partitions. One might imagine that the full-length sequence, incorporating all of the strongly discriminating subsequences should have superior discriminatory power. The issue is that a full-length sequence also incorporates those regions of the protein that are strongly conserved across all members of the family, regions that are conserved only in certain lineages as well as those which have little conservation. All of these regions will contribute to the overall homology scored by BLAST and tend to wash out the functional signal encoded by the partition. It is important to realize that SIMBAL does not reward conservation *per se*, but rather discrimination. A subsequence site may have little obvious conservation among the sequences of the desired class, but so long as its range of amino acids at particular sites is distinctly different from that of the rest of the partition, it will tend to be found. Additionally, full-length sequences may include structural differences (insertions and deletions) irrelevant to the functional differences underlying the partition. SIMBAL, like other motif-based approaches is insensitive these gross changes in protein length.

Methods such as INTREPID [21] have been developed to identify in multiple sequence alignments the critical sites that discriminate between branches of calculated phylogenetic trees. The approach presented here is independent of any multiple sequence alignment or tree calculation procedures, avoiding the possibility of errors they may introduce or the computational burden involved in their accurate generation. Similarly, SIMBAL does not involve any explicit training algorithms or parameters that must be tuned. Most importantly, SIMBAL requires neither painstaking compilation of experimentally verified training set data, nor assumptions about functional homogeneity in clades selected from larger protein families. SIMBAL's execution for a full scan at every window size scales with the square of the length of the object protein. Full scans will not usually be necessary (a limited range of window sizes from 5-30 residues suffices for most applications) allowing execution in linear time with protein length.

The method of mapping SIMBAL-identified hotspots onto crystal structures is used here to lend credence to our assertion that identified subsequences point to functional specificity. Finding the precise boundaries of these subsequences, that is, determining that one residue is critical but another nearby is not, is likely beyond the resolution of this method (due to the limitations of BLAST comparisons of short sequences). The algorithm is designed to mine information from noisy data sets, at the expense of single amino acid resolution. We note, however, that SIMBAL heat maps often show unambiguous peaks with pronounced edge effects, scores falling off sharply with single-residue shifts in subsequence length or location (for instance in Figure 6), from which one may predict the importance of a particular residue. Decisions as to what lengths of subsequences to map onto available crystal structures, will be determined by users in a case-by-case manner to illustrate emergent discoveries, rather than by the imposition of ad hoc rules. The development of ancillary tools to facilitate such graphical manipulation of these data sets will be beneficial.

Phylogenetic profiling, a discovery method in comparative genomics, has certain limitations. It identifies protein families correlated to some particular trait, and therefore provides an enrichment of proteins most likely to have a meaningful biological connection. But in the absence of secondary clues such as conserved operon structure, such hypothesized connections may offer only limited hints for explaining a protein's biochemical function and metabolic role. The method introduced here, SIMBAL, provides a means to continue investigations once profiling methods have generated first-round hypotheses. Phylogenetic profiling depends on substantial functional homogeneity within a protein family; high rates of neofunctionalization in a protein family may complicate use of the method by causing equivocal scores and hard-to-interpret results. By contrast, neofunctionalization events (mutations that change protein function) improve SIMBAL results (just as lateral transfer and gene loss events improve phylogenetic profiling), allowing the method to distinguish sequence differences that imply altered function from those that do not.

We suggest that, where results of profiling methods such as PPP appear equivocal, the following protocol may perform better than PPP alone. First PPP identifies good candidates for protein families co-distributed phylogenetically with some assigned trait, as in the example of ABC transporter permeases that score well based on a query profile of urea utilization. Next, an expansive set of homologs to each candidate protein is generated from completed genomes and partitioned according the trait, to serve as a training set. Finally, SIMBAL scans candidate proteins, and discovers if there are molecular signatures that outperform analyses based on the full-length protein only. SIMBAL extraction of suggested key motifs appears able to act as a "primitive", a basic operation that can identify multiple exemplars of protein subsequences important for functional prediction. We expect any number of downstream uses to become possible, such as building HMMs from SIMBAL-identified motifs at homologous positions, defining regular expressions to use in conjunction with other classifiers in functional annotation, or using SIMBAL to guide the creation of classifiers constructed from discontinuous signatures. Such SIMBAL-derived classifiers would be expected to outperform individual SIMBAL hot-spot sequences just as an HMM or PSI-BLAST model is expected to outperform BLAST based on a single sequence.

## Conclusions

In Partial Phylogenetic Profiling, the implicit "training set" is all proteins from all genomes in the TRUE partition of the profile. This training set is noisy of course - usually fewer than one protein in 1000 actually match the reference profile in a meaningful way - yet the power of profiling methods is beyond dispute. SIMBAL is likewise a discovery method based on efficient data mining after provision with a potentially noisy training set, where the training set now is the entirety of a protein family, partitioned according to some property calculated on each source genome. The method efficiently identifies prime candidate sites for conferring functional specificity to the proteins that contain them, and will be applicable in protein families where little or no direct characterization work has been done. The method will likely provide an excellent complement to protein crystallographic studies as a means to infer the importance of protein functional determinants, and provide mechanisms to develop improved protein functional classifiers for automated annotation systems.

## Methods

### Genome Properties Analysis

A phylogenetic profile for "urea utilization" was calculated by Genome Properties [4], based on complete genomes in the Comprehensive Microbial Resource, or CMR [11] having either of two component properties, "urease" http://cmr.jcvi.org/cgi-bin/CMR/shared/GenomePropDefinition.cgi?prop_acc=GenProp0051 or "urea carboxylase/allophanate hydrolase" [22]http://cmr.jcvi.org/cgi-bin/CMR/shared/GenomePropDefinition.cgi?prop_acc=GenProp0481. Prokaryotic species with fully sequenced genomes in which either urea utilization pathway scored at least 80% complete were entered in the profile as TRUE, and those with no markers for either system were entered as FALSE. The few remaining species with ambiguous results from metabolic reconstruction were not used.

### Partial Phylogenetic Profiling

Partial Phylogenetic Profiling (PPP) was performed as described previously [7], where precomputed BLAST search results were obtained from the CMR [11], a comparative genomics database for completed prokaryotic genomes. PPP is a method to query a genome according to a phylogenetic profile, that is, a list genomes designated TRUE or FALSE according to selection criteria such as occurrence of some marker gene. All proteins from the target genome are evaluated, in competition with each other, for how well their top sets of similar sequences by BLAST come preferentially from genomes marked as TRUE. For a given protein in the target genome, agreement with the profile is evaluated at all depths in its list of top-scoring BLAST matches, using the binomial distribution to find the depth where the odds of matching as well by chance are minimized. For different proteins in the target genome, the best match to the profile occurs at different BLAST score cutoffs and different depths into their respective lists of best hits, and the depth is optimized on the fly rather than through pre-set cutoffs. PPP scores are reported as the (maximized) negative log likelihood that such a preponderance of TRUE hits over FALSE hits might have occurred by chance given the ratio of TRUE-labeled to FALSE-labeled proteins in the partitioned BLAST database.

### Training set construction

SIMBAL requires two sequence collections derived from a larger homology family that differ in some asserted attribute. Two methods were used in this study (Figure 2) and are described below, but any number of alternatives may be explored by those wishing to implement this technique, depending on the nature of the system being studied.

#### Example 1

Possible urea ABC transporter permease subunits were partitioned according to their respective "urea utilization" metabolic backgrounds. A set of candidate permease subunits for urea uptake transporters was generated from CMR proteins matching PF02653 [10], which includes the two permease subunits (UrtB and UrtC) of the *Corynebacterium glutamicum *urea transporter operons [11], plus all CMR proteins matched by other members of the same Pfam clan, CL0142: PF00950, PF01032, PF01098, and PF05145. This set totals 10,111 sequences, the majority of which are not urea transporters. These proteins were partitioned according to whether (TRUE) or not (FALSE) the genome of origin encodes a urea utilization system as determined by Genome Properties analysis (see above). Note that this method naively places many non-urea permeases in the TRUE set in addition, modeling the situation where no other corroborating evidence exists.

#### Example 2

TIGRFAMs model TIGR00536 describes a family of S-adenosylmethionine (SAM)-dependent protein methyltransferase proteins that includes PrmC (HemK) [19], PrmB [23], and others that are uncharacterized. Putative methylases recognized by TIGR00536 were partitioned according to proximity to the *prfA *gene as recognized by TIGR00019, encoding the peptide chain release factor 1 protein (RF-1), a substrate of PrmC. TIGR00536-family methylases observed no more than two genes away from *prfA *were assigned to the TRUE partition. Those found as the second or third paralog in a genome, where the first was near *prfA*, were assigned to the FALSE partition. TIGR00536-family methyases in genomes where no member is in close proximity to the *prfA *gene were excluded from the analysis.

For each training set, the TRUE and FALSE partitions separately were made non-redundant at the level of 80% sequence identity with preferential removal of fragmentary proteins. The resulting sequence sets were then analyzed by SIMBAL.

### Site-profiling by SIMBAL

SIMBAL (Sites Inferred from Metabolic Background Labels) is performed by the program SIMBAL.pl, written in Perl. Inputs to the program include a query sequence, a file of proteins for the TRUE partition of the training set, and a file of proteins for the FALSE partition. SIMBAL.pl produces a combined sequence library in which proteins are labeled according to their partition of origin and performs BLAST searches for subsequences of user-specified size ranges. Access to SIMBAL.pl and release notes are provided, and sample TRUE and FALSE data sets are available for download at http://www.jcvi.org/openAccess/uploadSimbalForm.html. SIMBAL.pl produces tab-delimited output. A web resource that uses the tabular output to draw triangular heat maps (as in Figure 3) is found at http://www.jcvi.org/openAccess/simbalPlotViewer.html. SIMBAL.pl source code is freely available by request.

For each sub-sequence of the query protein, taken in turn, the list of BLAST hits to the training set is analyzed at increasing depths in the hits list. SIMBAL analysis for the examples presented here were performed in single-hit mode, where only the first sequence encountered per genome from the TRUE partition counts in scoring, although all hits to the FALSE partition count (a multi-hit mode is supported in the program, for use in cases where numerous genes in the TRUE genomes are expected to carry the trait in question). The SIMBAL score at each depth represents the log odds against the actual number (or greater) of sequences labeled TRUE occurring at that depth by random chance, and the reported score for each subsequence is the common log of the optimal (lowest) score from any depth. As in PPP, this statistic is calculated using the binomial distribution [7]. SIMBAL reports high significance for subsequences whose top BLAST matches are dominated by large numbers of hits to sequences from the TRUE branch of the partition. It reports low significance for all subsequences where the top-scoring matches by BLAST show no bias toward sequences labeled TRUE, and also where BLAST finds few hits of any kind, as happens for short subsequences from poorly conserved regions.

Note that the sequences as evaluated by Partial Phylogenetic Profiling are full-length, and BLAST is (to a first approximation) sensitive and specific enough to detect relevant homologs out of all proteins from all current complete genomes. SIMBAL, in contrast to PPP, searches with subsequences as short as 5 amino acids, making its BLAST searches far more sensitive to noise from spurious matches. So while the implicit training set for PPP consists of all proteins from all genomes in the profile (divided into TRUE and FALSE partitions), the explicitly constructed training set for SIMBAL is restricted to one family of proteins. The expedient of running BLAST searches against a limited set of homologs rather than against all proteins should cause no loss of information. SIMBAL is only meant to be applied to members of a particular homology family, thus, BLAST hits to its subsequences outside of that family cannot be considered indicators of a functional correlation. Indeed, such hits, especially for short sequences, are likely to be due to noise. Instead, the relatively small size of the training set provides both better search specificity and a great advantage in terms of the speed of the algorithm.

Using our web service, which runs each job on a single 2.66 GHz Intel Xeon processor, versus a typical protein sequence of 300 amino acids, execution time averages approximately one second for each subsequence analyzed for a full scan. Thus, as an upper limit, using a 1 amino acid window shift and checking every subsequence size from 10 to 300, a complete scan would execute in somewhat less than 12 hours. Such a scan, however, would only need to be done to create manuscript-quality triangle figures such as the ones shown in figures 3 and 6. In a typical usage, a rougher initial scan can be performed with a window shift of 4, a subsequence length jump of 4 and an upper subsequence length limit of 40 amino acids. Such a scan executes in only 3 minutes.

Scores produced by SIMBAL, like those from PPP, are uncalibrated and should not be interpreted as reporting actual statistical significances, but rather are to be used for comparison purposes only. The species most closely related to each other tend to have the most similarity in gene content, and the highest levels of sequence identity across orthologous pairs. Consequently, many subsequences from numerous proteins examined by SIMBAL will tend toward matching the "true positive" partition of the training set preferentially, even if the subsequence in question has no direct bearing on the trait in question. Meaningful signal, however, will stand out from this background by multiple orders of magnitude. SIMBAL can use the binomial distribution naively, as if each genome arises independently, uncomplicated by taxonomic relationships, because the method is used to generate comparative rather than absolute scores.

## Abbreviations

PPP: Partial Phylogenetic Profiling; SIMBAL: Sites Inferred from Metabolic Assertion Labeling; CMR: Comprehensive Microbial Resource; HMM: Hidden Markov Model.

## Authors' contributions

JDS and DHH contributed equally to the algorithmic development and basic research represented by this manuscript as well as its preparation. DBR provided support through the development of software and web site construction as well as critical commentary on the manuscript.
